# Supplementary material for: Climate drivers and palaeobiogeography of lagerpetids and early pterosaurs
Source: Nat Ecol Evol. 2025 Jun 18;9(8):1359–72. doi: 10.1038/s41559-025-02767-8 (PMC12328205; doi:10.1038/s41559-025-02767-8)
Supplement: Supplementary file 1 — Supplementary discussion, Figs. 1–3, Tables 1 and 2 and references. [file 41559_2025_2767_MOESM1_ESM.pdf]

nature ecology & evolution

Supplementary information https://doi.org/10.1038/s41559-025-02767-8

Climate drivers and palaeobiogeography of lagerpetids and early pterosaurs

In the format provided by the authors and unedited

Table of contents

- Supplementary Discussion
    - Pterosauromorpha fossil record
    - Comparison with Müller et al. (2023)
    - Palaeoclimate model simulation uncertainties
    - Simulation evaluation
- Supplementary Tables
- Supplementary References

Supplementary Discussion

The fossil record of Triassic pterosauromorphs

Within Pterosauromorpha, lagerpetids have a wide latitudinal and geographic spread through most of their evolutionary history (late Ladinian–late Norian, 237–211 Ma). *Kongonaphon kely*, the oldest known lagerpetid, is from the Ladinian–Carnian aged Makay Formation ("basal Isalo II beds") of Madagascar (Kammerer et al. 2020). Other Carnian lagerpetids include: *Scleromochlus taylori* from the Lossiemouth Sandstone Formation of Scotland, UK (Woodward 1907; Benton 1999; Bennett 2020; Foffa et al. 2022, 2023); *Lagerpeton chanarensis* from the Chañares Formation of Argentina (Romer 1971; Sereno and Arcucci 1993); an unnamed lagerpetid (UFSM 11611) from equivalent layers of the middle portion of the Candelária Sequence/upper portion of the Santa Maria Formation of southern Brazil (*Hyperodapedon* Assemblage Zone, mid-Carnian, Langer et al. 2018) (Garcia et al. 2019); and the sympatric *Ixalerpeton polesinensis* and *Venetoraptor gassenae* from the Santa Maria Formation of Brazil (Cabreira et al. 2016; Müller et al. 2023). It is only from the Norian that lagerpetids (i.e., *Dromomeron romeri*, *Dr. gregorii*) are found at low latitudes (southwest USA, Chinle Formation and Dockum Group: Irmis et al. 2007; Nesbitt et al. 2009), while maintaining a constant presence in high-latitude Pangaea (i.e., *Dromomeron gigas*, PSVJ 883) (Argentina: Martínez et al. 2012, 2016; Müller et al. 2018). With the exception of fragmentary, and largely undescribed specimens from the early-middle Norian Dockum Group (Tecovas Formation) and Chinle Formation (Blue Mesa Member), the earliest pterosaurs (i.e., *Eudimorphodon ranzi*, *Seazzadactylus venieri*, *Peteinosaurus zambellii*, among others) are found in middle Norian marine deposits around the low latitude Tethyan margins (Zambelli 1973; Wild 1978; Dalla Vecchia 2013, 2019; Baron 2021). From the late Norian, pterosaur fossils are found in a wider variety of habitats, ranging from low-latitude coastal and desert, to high latitude fluvial and continental settings of the Tethys Gulf, the southwestern USA, South America, and the Rhaetian of Greenland (Jenkins et al., 2001; Dalla Vecchia 2013; Andres et al. 2014; Kellner 2015; Upchurch et al. 2015; Britt et al. 2018; Kellner et al. 2022; Baron 2021; Martínez et al. 2022). The abrupt ecological and geographical spread of early pterosaurs provides evidence of serious sampling biases (Butler et al. 2009, 2011; 2013; Dean et al. 2016), but might also imply that it was a genuinely fast evolving, fast spreading clade (Yu et al. 2023). Still, sympatric occurrences of lagerpetids and pterosaurs are rare during their ~20 million-year Norian–Rhaetian stratigraphic overlap with examples limited to the Quebrada del Barro Formation (*Dromomeron gigas* and *Yelaphomte praderioi*; Martínez et al. 2014, 2022) and a single locality in the Norian southwestern USA (PFV 456, Thunderstorm Ridge).

The fossil record of pterosauromorphs is, like all other groups, incomplete. The majority of Triassic pterosauromorph fossils have been sampled from parts of Northern America, South America, and Europe (Supplementary Figure 1). This global distribution broadly reflects extensively documented processes that generate heterogeneity of the fossil record, such as those related to taphonomy (preservation), geographical location, research effort, and socio-economics (Raup 1972; Dean et al. 2016; Benson et al. 2021; Raja et al. 2021). For pterosaurs specifically, their distribution correlates with the locations of Lagerstätten throughout the Mesozoic (Dean et al. 2016). Interestingly, while the pterosauromorph fossil record is patchy, there are several extensively-studied locations where pterosauromorph taxa have not been sampled, indicating that the known fossil record is at least somewhat reflective of true distributions and, by extension, environmental preferences. In our study, we attempted to mitigate several issues associated with heterogeneous sampling of the fossil record. First, we applied a specimen-based approach that included all available occurrences of Triassic pterosaurs (including fragmentary, undescribed and indeterminate specimens). This strategy was implemented for the palaeoclimate niche occupation analyses and the habitat suitability models. This permitted additional spatial and temporal data points to be included in these analyses, thus resulting in the most comprehensive examination of palaeoclimate niche occupation and habitat suitability modelling currently possible for this taxonomic group.

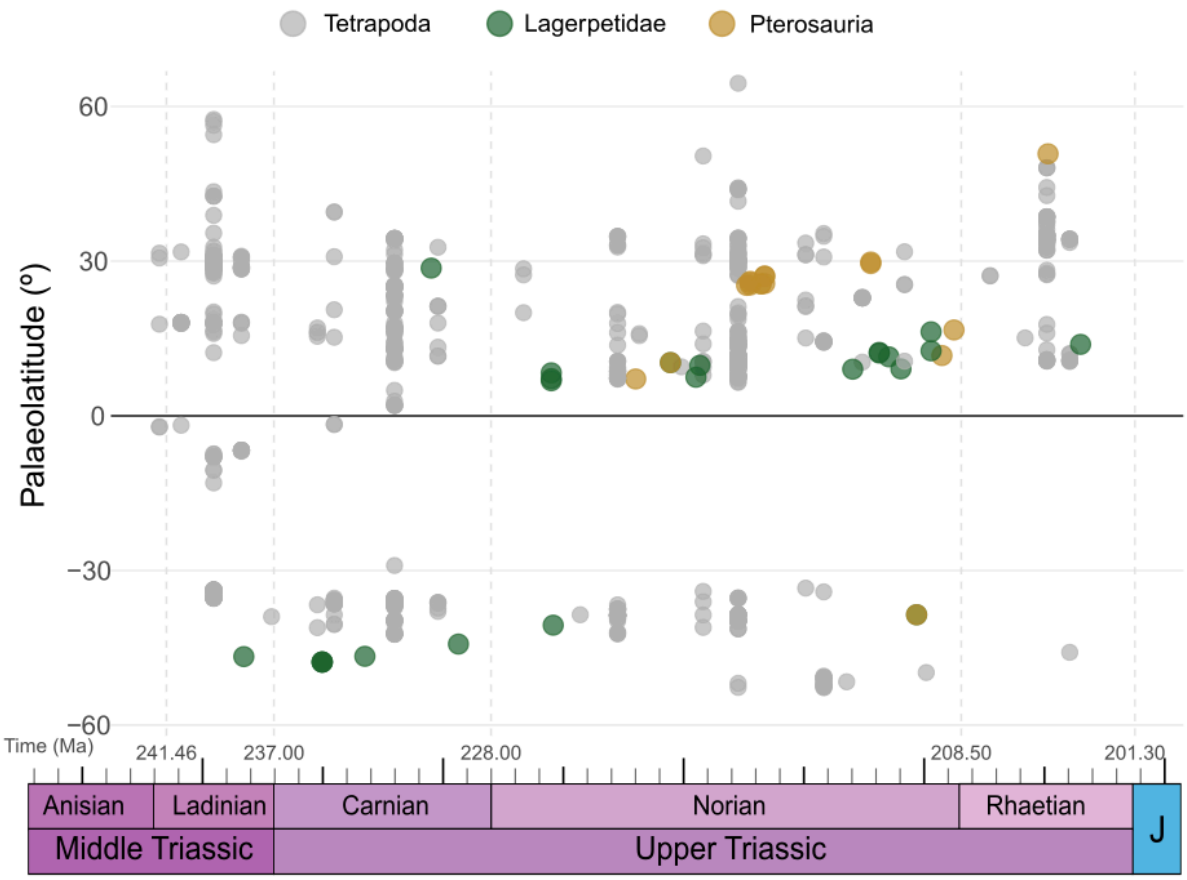

Supplementary Figure 1. Age and palaeolatitudinal occurrences of Middle and Late Triassic tetrapod bearing locations.

The previously described specimen-based approach is not possible for the historical biogeographic analyses that rely on phylogenetic data, where fragmentary and undiagnostic specimens cannot be included. Our phylogeny samples the most complete pterosauromorph sample available from the literature. Nevertheless, it does not include some taxa of ambiguous affinities (e.g. *Arcticodactylus*) and a handful of indeterminate occurrences that we used in the palaeoclimate-based analyses (e.g., MCSNB 8950, PEFO 53384, SMU 69125, PEFO 45782 amongst pterosaurs; and PEFO 50545, UFSM 11625, PEFO 44476, PEFO 50545 amongst lagerpetids). In these circumstances, it is often possible to graft absent taxa into a tree when their affinities are not sufficiently established. This is not possible in this case. For example, irreconcilable uncertainties regarding the phylogenetic position of *Arcticodactylus* made it impossible to include this taxon in the phylogenetic dataset. Specifically, *Arcticodactylus* was hypothesised to be a eudomorphodontid closely related to *Eudimorphodon* and *Carniadactylus* (Upchurch et al. 2020), but also in various other positions in close relationships with *Carniadactylus*, *Raeticodactylus*, or *Austriadraco* and *Seazzadactylus*, MCSNB 8950, depending on the analysis settings (Baron 2020). Similarly, we were unable to graft MCSNB 8950 into our tree topology with sufficient

certainty. A thorough review of the anatomy and ontogeny of these taxa will lead to a better understanding of their phylogenetic positions shortly.

In the meantime, we followed the strategy of Griffin et al. (2022) and randomly selected a single topology to run the biogeographical analyses. This strategy was demonstrated as a suitable solution in a situation where different datasets are available or a fully resolved strict consensus (SC) cannot be obtained (Griffin et al. 2022). Griffin et al. (2022) already showed the limited impact of changing archosaur topology on their results.

Finally, we report that an alternative phylogenetic topology for pterosauromorphs was hypothesised recently (Garcia and Müller 2025). This study proposes that Lagerpetidae is paraphyletic grade of taxa on the 'stem' of Pterosauria, within a monophyletic Ornithodira composed of *Faxinalipterus* and the clade Pterosauromorpha + Dinosauromorpha. If confirmed, this phylogenetic arrangement would fill in the ghost lineage between pterosaurs and their closest kin. However, by the admission of the authors, this hypothesis is preliminary and further testing is necessary to support this topology. In particular, a more complete sample of lagerpetids (including *Scleromochlus*) and pterosaurs (only five were scored in the study) should be included in future iterations of the analysis. For its current incompleteness and until further testing, we decided against using this topology in our analysis.

Comparison with Müller et al. (2023)

The latitudinal dispersal pattern of dinosaurs recovered by our analyses is much lower than that presented by Müller et al. (2023). This difference is, once again, explained by different sampling strategies. The increased sampling of dinosaur taxa in our dataset also increased the number of dispersal events. This has two effects: first, it increases the range of values captured by the non-standardised curve (by increasing the accumulated degrees of dispersal); and second it lowers the standardised curve (by increasing the dividend in the standardisation formula). Nonetheless, the overall pattern is maintained: dinosaur accumulated degrees of latitudinal dispersal (standardised or not) peaks in the first half of the Norian, before plateauing through the rest of the Norian (Fig. 2b–c). The consistency of this pattern in two largely independent datasets, speaks to its robusticity. There is an absolute and relative increase of accumulated dispersal towards the end of the Rhaetian, which was not recorded by Müller et al. (2023) and does not correspond to the increased potential for dispersion presented by Griffin et al. (2022). This can be explained by two factors: first, this section of our dinosaur phylogeny is rich in dispersal events that were not recorded in the smaller sample-size of the Müller et al. (2023) dataset. As we have seen above, expanding the datasets, particularly with Jurassic dinosaurs, leads to a higher

number of dispersal events and thus raises the curve; second, crucially, these events almost exclusively occur outside of climatic barriers, and thus are not picked up by our original models (Fig. 2a).

Palaeoclimate model simulation uncertainties

Databases of global climate proxy data are available for past time periods; however, proxy-evidence becomes increasingly less constrained, and the amount of spatiotemporal data diminishes, increasingly further into the geologic past. This necessitates the use of palaeoclimate model simulations, to provide global coverage. However, palaeoclimate model simulations of deep-time climates are challenging, and themselves have associated uncertainties. These uncertainties can in general be partitioned into two main sources, i) boundary condition uncertainty, and ii) global climate model uncertainty.

(i) Boundary condition uncertainty.

Palaeoclimate model simulations require certain boundary conditions to be prescribed by the user. The most important boundary conditions are (a) palaeogeographic reconstructions, (b) ice sheet height and extent, (c) solar luminosity, (e) orbital configuration, and (e) greenhouse gas concentrations.

(a) Palaeogeographic reconstructions, or Digital Elevation Models (DEMs), derived from paleo data relating to elevation and plate movements, provide orography, bathymetry, and land-sea distribution. These are crucial for determining local, regional, and global atmospheric and ocean circulation and as a result the underlying climate produced by the model. The further back we go into the geologic record, the less these DEMs are constrained by data, which in turn creates larger uncertainties in these reconstructions. However, our understanding of plate tectonics, spreading ridges, weathering rates and basinal deposition allows, in general, an accurate first order approximation of deep-time palaeogeography. The largest uncertainties usually result from the height and depth of topographic and bathymetric surfaces and their spatial coverage (e.g. Farnsworth et al. 2021). For this study, we use the PALEOMAP palaeogeographic reconstruction (Scotese and Wright, 2014).

(b) Ice sheets (and associated sea-level) can have a large impact not only on regional climate but also global climate, due primarily to changes in land surface area, surface albedo, and changes in surface height. The HadCM3L model used in this study requires the height and extent of ice sheets to be prescribed. For the Triassic there were no known large land ice sheets due to surface temperatures being too warm because of high pCO_2 concentrations (>1000 ppm). As such, the paleoclimate model simulations

used here do not have any ice sheets prescribed, removing one source of potential uncertainty.

(c) Solar luminosity, the energy flux from the Sun received by the Earth at the top of the atmosphere, is relatively well known. Gough (1981) approximated the temporal evolution of solar luminosity based on a simple linear model based on the age of the parent star. Apart from the first 0.2 Gyr this approximation is shown to agree well with observations (Bahcall, et al. 2001). As such, the uncertainty associated with solar luminosity in the Triassic simulations in this study is relatively low.

(d) Orbital configuration, the eccentricity, obliquity, and precession of the Earth's orbit around the Sun can have a substantial impact on the seasonal climate signal, which, in turn, can lead to significant changes in climate state (e.g. Pleistocene glacial/interglacial cycles). This is due to ice sheet formation and melt, impacts on the carbon cycle leading to changes in atmospheric CO_2, and associated changes in global atmospheric and oceanic configuration. Often for deep-time simulations a modern orbital configuration is imposed. This is because chronological uncertainty in the proxies we wish to compare the model against will often cover many orbital cycles. This may result in the proxy record being more representative of a mean orbital state (which is similar to that of the modern day, due to the relatively low modern eccentricity). This is particularly the case here, where our seven time periods are separated by tens of millions of years, for comparison with 20,000 year precession-related orbital cycles (see also Kent and Olsen 1997).

(e) Greenhouse gas concentrations, more specifically, pCO_2 concentrations, vary through the geologic past, due to long-term imbalances between tectonic emissions and weathering. CO_2 concentrations can be estimated from CO_2 proxies, but proxy type, age, techniques, and calibration uncertainty, as well as temporal sparsity of records, can all make constraining a deep time pCO_2 concentration problematic. Here we use the CO_2 record of Foster et al. (2017), which accounts for some of the issues above, but is still associated with substantial uncertainties.

(ii) Global model uncertainty. Although all global climate models use the same well-known sets of equations to simulate the behaviour of the Earth system, results can vary between models, in particular at local and regional scales. This is largely due to the fact that many processes in the climate system occur at a spatial scale smaller than the model resolution, requiring approximations (parameterisations) to be used to represent processes such as cloud formation and development.

However, confidence in the robustness of our results can be obtained by the facts that: (a) the HadCM3 family of models, although 20 years older than many of its

contemporaries, has contributed to the Fifth Coupled Model Intercomparison Model () five, and shows similar skill to many other CMIP5 models in its representation of the modern climate (Valdes et al. 2017). (b) HadCM3L-M2.1D has seen continued development (Valdes et al. 2017, Valdes et al 2021). Here we use an updated version of the model which solves a persistent problem with the majority of paleoclimate model known as the 'cold pole paradox' where the simulated higher latitudes were previously much cooler than suggested by proxy-observations, by using methods similar to those in Sagoo et al. (2014) and Kiehl and Shields (2013). (c) These simulations for the Induan–Maastrichtian interval have been run out for over 9,000 model years. Often, palaeoclimate simulations are run for only a relatively short period due to computational costs. However, it can take upward of 5,000 years to truly allow a model simulation to equilibrate to all the applied model forcings, in particular for the deep ocean, and as such, the global ocean circulation to be fully representative of the deep-time period. (d) Although model uncertainty is important to constrain it has been shown, at least for future climate simulations, that scenario uncertainty, in other words the applied boundary conditions, is the largest source of error (Hawkins and Sutton 2009).

Simulation evaluation

Simulation evaluation against available palaeoclimate proxy evidence is not a straightforward task. This is because proxy data are associated with considerable uncertainty since they are by nature indirect and are often unconstrained temporally and because they can be relatively sparse spatially. Here, we compare lithological indicators of climate with our climate model outputs. In particular, we compare geological evidence for coal deposits with regions of potential deposition of coal deposits as predicted by the climate model.

For the geological evidence for coal deposits, we use the Upper Triassic Atlas of Lithological Indicators of Climate (Boucot et al. 2013), which synthesizes 430 lithological indicators in the Upper Triassic. Here, we make use of the indicators of coal deposits that are well enough dated to be categorised at a Stage level. This gives 63 coal records for the Carnian, 93 for the Norian, and 104 for the Rhaetian. All sites are rotated to their palaeolatitudes and longitudes using a reference frame consistent with the model simulation of that Stage. For the model-predicted potential coal deposits, we use methods outlined in Craggs (2012), which is based on the modeled mean annual temperature and precipitation and seasonality. Values >50% typically signify a region where coal/peat formation could potentially occur. As such, this provides an evaluation of both the temperature and precipitation from the climate model.

Overall, for all the simulations, known coal deposits in general match well with those regions predicted by the six Upper Triassic model simulations, i.e. where the model-derived coal formation potential is >50% (Supplementary Figure 2). In addition, Xie et al. (2024) carry out a point-point comparison of the model-predicted evaporites with sedimentological proxies. This can be considered a test of the hydrological cycle in the model. It is not for the Upper Triassic specifically, but for four snapshots in the Phanerozoic, one of which is at 252 Ma. The paper concludes that there is "reasonable agreement" with the proxies.

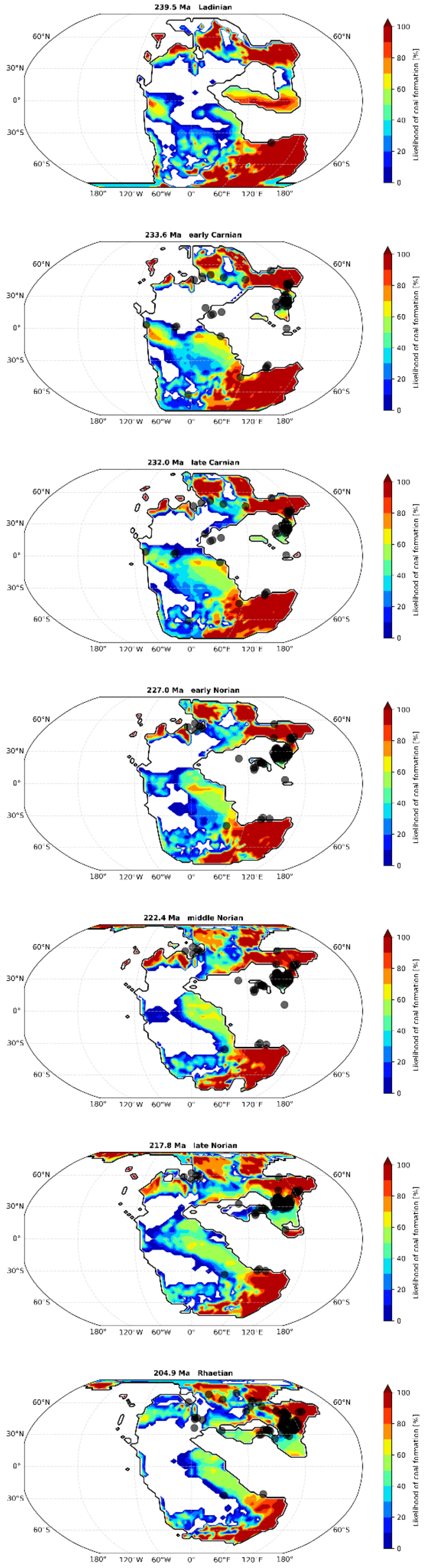

Supplementary Figure 2. Predicted model coal locations (%) from the six Upper Triassic paleoclimate model simulations and known coal locations during the Upper Triassic (black dots). The potential to form coal and peat (represented by the blue to red gradient) in the model is based on mean annual temperature, precipitation and seasonality (Craggs et al. 2012). Values >50% typically characterise regions where coal/peat formation can occur.

In addition, the Carnian Pluvial Episode (CPE; ~232–230 Ma) was an interval that departed from the overall dry climate of the Triassic as a whole (Ruffell et al. 2016), shifting into a more humid climate throughout the continent. Deposits associated with humid conditions, such as coals, peats, fluvial sandstones, mudstones and other deposits suggestive of a more intense hydrological cycle have been found to be widely distributed throughout most of Pangaea at this time. The strongest evidence for such a humid period can be seen in coal deposits (Figure S2, black dots). Known palaeo-rotated coal deposits match well with the 232 Ma Carnian simulation depicting model-derived coal formation potential (>50%; Fig. S2).

Further evidence comes from humid deposits (Ruffell et al. 2015) corresponding well with regions of warm, wet conditions and humid conditions (annually/seasonally) in the model (Supplementary Figure 3). Arid climates typically receive less than 250 mm/yr (>0.7 mm/day) precipitation. Only three small regions of the continent have less precipitation which constitutes an arid climate (northwest, western equatorial region, and western southern mid-latitudes).

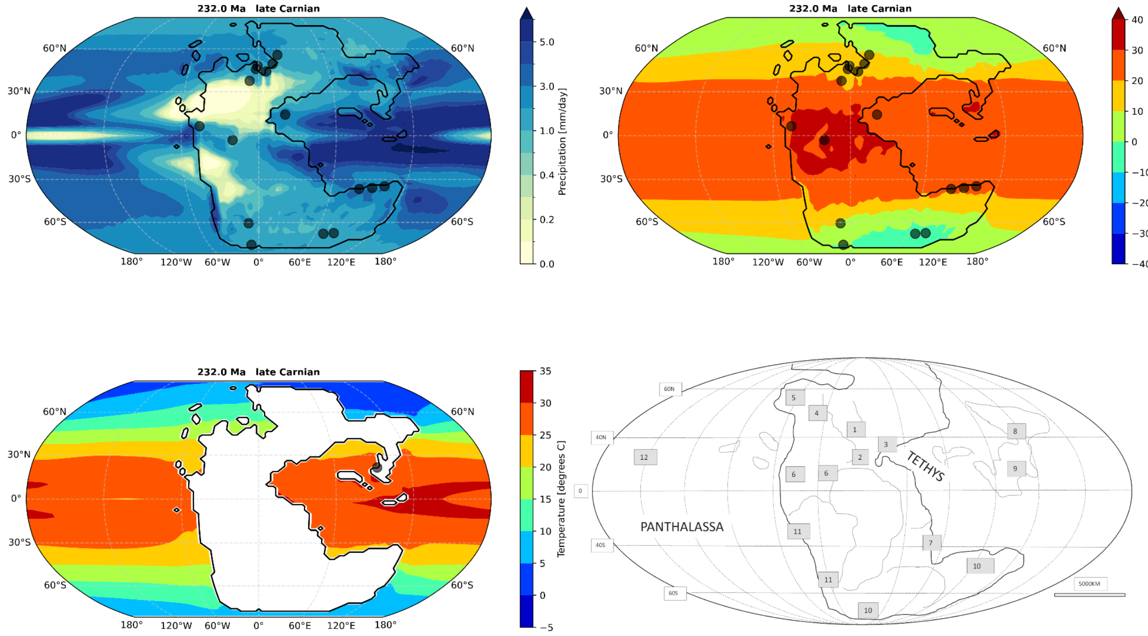

Supplementary Figure 3. Carnian (232 Ma) paleoclimate model outputs. Annual mean precipitation (mm/day; top left), 1.5m air temperature (°C; top right). Black circles represent known paleo-rotated coal deposits. Sea surface temperatures (°C; bottom left). 'Black dot' shows proxy-SST location (Sun et al. 2016). Shown against humid deposits for the late Carnian (bottom right; reproduced from Ruffell et al. 2015).

Direct comparisons between geochemical proxies and Global Circulation Models (CGM) are rare for deep time studies, but some exist. These show that the estimation of climate variables extracted from sedimentological, geochemical, and biological proxies are consistent with CGM predictions at basin level. For example, Mancuso and colleagues

(2021, 2022) demonstrated that multiproxy from clay mineral geochemical data estimates for mean annual temperature precipitation and high-temperature seasonality from the Chañares-Los Rastros-Ischigualasto succession (early Carnian to early Norian) match the predictions of the GCM used by Dunne et al. (2021), which is a predecessor of that we used here. These observations are still valid with the current version of the GCM.

The palaeoclimate of this basin fluctuated from warmer, drier conditions to more temperate humid conditions in the early Carnian to early Norian interval, as broadly predicted by our GCM (see Extended Data Fig. 4). Similarly, Lepre and Olson (2020) used sedimentological and geochemical data to demonstrate a trend of increasing aridity and lower precipitation in the Colorado Plateau Chinle Formation succession (early Norian to Rhaetian). The same trend was recovered by our GCM (see Extended Data Fig. 4). Climate prediction from GCM also corresponds well with inferred Triassic floral provinces (Kustatscher et al., 2018; Mancuso et al. 2021).

Supplementary Tables

Supplementary Table 1. Comparisons of palaeobiogeographical models.

MODEL	AIC
DEC	622.8216
DECj	404.4480
LIKEDIVA	645.4061
LIKEDIVAj	403.1796
BAYAREALIKE	699.2676
BAYAREALIKEj	701.2869

Supplementary Table 2. PCA scores and character weights.

	PC1 (57.2%)	PC2 (41.6%)	PC3 (4.8%)	PC4 (1.0%)
**MAT (°C)**	0.3894	-0.5971	-0.6707	0.2050
**MAP (mm/day)**	0.4125	0.6117	-0.1011	0.6674
**Seasonal temperature**	-0.5463	0.4038	-0.7200	-0.1415
**Seasonal precipitation**	0.6162	0.3259	-0.1469	-0.7018

Supplementary References

Farnsworth, A., Valdes, P.J., Spicer, R.A., Ding, L., Witkowski, C., Lauretano, V., Su, T., Li, S., Li, S. and Zhou, Z. (2021). Paleoclimate model-derived thermal lapse rates: Towards increasing precision in paleoaltimetry studies. *Earth and Planetary Science Letters*, **564**, p.116903.

Bahcall, J.N., Pinsonneault, M.H. and Basu, S. (2001). Solar models: Current epoch and time dependences, neutrinos, and helioseismological properties. *The Astrophysical Journal*, **555**(2), p.990.

Benson, R. B., Butler, R., Close, R. A., Saupe, E., and Rabosky, D. L. (2021). Biodiversity across space and time in the fossil record. *Current Biology*, **31**(19), 1225–1236.

Boucot, A.J., Xu, C., Scotese, C.R., and Morley, R. J. (2013). *Phanerozoic paleoclimate: an atlas of lithologic indicators of climate* **11**, 1–30. Tulsa, OK: SEPM (Society for Sedimentary Geology).

Craggs, H., Valdes, P.J. and Widdowson, M. (2012). Climate model predictions for the latest Cretaceous: an evaluation using climatically sensitive sediments as proxy indicators. *Palaeogeography, Palaeoclimatology, Palaeoecology*, **315** 12–23. https://doi.org/10.1016/j.palaeo.2011.11.004

Garcia, M.S., and Müller, R. T. (2025). Triassic pterosaur precursors of Brazil: catalog, evolutionary context, and a new hypothesis for phylogenetic relationships of Pterosauromorpha. *Anais da Academia Brasileira de Ciências,* **97**(suppl 1), e20240844.

Hawkins, E., & Sutton, R. (2009). The potential to narrow uncertainty in regional climate predictions. *Bulletin of the American Meteorological Society*, 90(8), 1095-1108.

Kiehl, J.T. and Shields, C.A. (2013) Sensitivity of the Palaeocene–Eocene Thermal Maximum climate to cloud properties.*Philosophical Transactions of the Royal Society A* **371**, 20130093. http://doi.org/10.1098/rsta.2013.0093.

Kustatscher, E., Ash, S.R., Karasev, E., Pott, C., Vajda, V., Yu, J., and McLoughlin, S. (2018). *Flora of the late Triassic*. The Late Triassic World: earth in a time of transition, 545-622.

Lepre, C.J., and Olsen, P.E. (2021). Hematite reconstruction of Late Triassic hydroclimate over the Colorado Plateau. *Proceedings of the National Academy of Sciences*, 118(7), e2004343118.

Raja, N.B., Dunne, E.M., Matiwane, A., Khan, T.M., Nätscher, P.S., Ghilardi, A.M., and Chattopadhyay, D. (2022). Colonial history and global economics distort our understanding of deep-time biodiversity. *Nature Ecology & Evolution*, **6**(2), 145–154.

Raup, D.M. (1972). Taxonomic Diversity during the Phanerozoic: The increase in the number of marine species since the Paleozoic may be more apparent than real. *Science*, **177**(4054), 1065–1071.

Ruffell, A., Simms, M.J. & Wignall, P.B. (2015). The Carnian Humid Episode of the late Triassic: a review. *Geological Magazine*, **153**, 271–284.

Sun, Y.D., Wignall, P.B. et al. (2016). Climate warming, euxinia and carbon isotope perturbations during the Carnian (Triassic) Crisis in South China. Earth and Planetary Science Letters, **444**, 88–100.

Sagoo, N., Valdes, P., Flecker, R. and Gregoire, L.J. (2013) The Early Eocene equable climate problem: can perturbations of climate model parameters identify possible solutions? *Phil. Trans. R. Soc.* A. **371**, 20130123. http://doi.org/10.1098/rsta.2013.0123.

Xie, Y., Lunt, D.J., and Valdes, P.J. (2024) Diagnosing the controls on desert dust emissions through the Phanerozoic. *Clim. Past*, **20**, 2561–2585, https://doi.org/10.5194/cp-20-2561-2024
