# Supplementary material for: Climate drivers and palaeobiogeography of lagerpetids and early pterosaurs
Source: Nat Ecol Evol. 2025 Jun 18;9(8):1359–72. doi: 10.1038/s41559-025-02767-8 (PMC12328205; doi:10.1038/s41559-025-02767-8)
Supplement: Supplementary file 2 — Reporting Summary [file 41559_2025_2767_MOESM2_ESM.pdf]

nature portfolio

Corresponding author(s): Davide Foffa
Last updated by author(s): May 15, 2025

Reporting Summary

Nature Portfolio wishes to improve the reproducibility of the work that we publish. This form provides structure for consistency and transparency in reporting. For further information on Nature Portfolio policies, see our Editorial Policies and the Editorial Policy Checklist.

Statistics

For all statistical analyses, confirm that the following items are present in the figure legend, table legend, main text, or Methods section.

n/a	Confirmed	
☐	☒	The exact sample size (*n*) for each experimental group/condition, given as a discrete number and unit of measurement
☒	☐	A statement on whether measurements were taken from distinct samples or whether the same sample was measured repeatedly
☐	☒	The statistical test(s) used AND whether they are one- or two-sided<br>*Only common tests should be described solely by name; describe more complex techniques in the Methods section.*
☐	☒	A description of all covariates tested
☒	☐	A description of any assumptions or corrections, such as tests of normality and adjustment for multiple comparisons
☒	☐	A full description of the statistical parameters including central tendency (e.g. means) or other basic estimates (e.g. regression coefficient) AND variation (e.g. standard deviation) or associated estimates of uncertainty (e.g. confidence intervals)
☐	☒	For null hypothesis testing, the test statistic (e.g. *F*, *t*, *r*) with confidence intervals, effect sizes, degrees of freedom and *P* value noted<br>*Give P values as exact values whenever suitable.*
☒	☐	For Bayesian analysis, information on the choice of priors and Markov chain Monte Carlo settings
☒	☐	For hierarchical and complex designs, identification of the appropriate level for tests and full reporting of outcomes
☒	☐	Estimates of effect sizes (e.g. Cohen's *d*, Pearson's *r*), indicating how they were calculated

Our web collection on statistics for biologists contains articles on many of the points above.

Software and code

Policy information about availability of computer code

Data collection	The first iteration of occurrence data was done using the Palaeobiology Database. This first search proved inadequate for the scope of the study and was updated and expanded through literature search by DF.<br>No other software was used to collect data.
Data analysis	All analyses were performed using freely available software, and packages in R / R studio. Custom codes and data will be made available in a specified repository (figshare), alongisde pertinent data)

For manuscripts utilizing custom algorithms or software that are central to the research but not yet described in published literature, software must be made available to editors and reviewers. We strongly encourage code deposition in a community repository (e.g. GitHub). See the Nature Portfolio guidelines for submitting code & software for further information.

Data

Policy information about availability of data

All manuscripts must include a data availability statement. This statement should provide the following information, where applicable:

- Accession codes, unique identifiers, or web links for publicly available datasets
- A description of any restrictions on data availability
- For clinical datasets or third party data, please ensure that the statement adheres to our policy

Data have been made available to reviewers and will be made available upon accepance in a specified repository (figshare).

Research involving human participants, their data, or biological material

Policy information about studies with human participants or human data. See also policy information about sex, gender (identity/presentation), and sexual orientation and race, ethnicity and racism.

Reporting on sex and gender	n/a
Reporting on race, ethnicity, or other socially relevant groupings	n/a
Population characteristics	n/a
Recruitment	n/a
Ethics oversight	n/a

Note that full information on the approval of the study protocol must also be provided in the manuscript.

Field-specific reporting

Please select the one below that is the best fit for your research. If you are not sure, read the appropriate sections before making your selection.

☐ Life sciences ☐ Behavioural & social sciences ☒ Ecological, evolutionary & environmental sciences

For a reference copy of the document with all sections, see nature.com/documents/nr-reporting-summary-flat.pdf

Ecological, evolutionary & environmental sciences study design

All studies must disclose on these points even when the disclosure is negative.

Study description	This study is 2-fold:<br>1) Palaeobiogeography. This section utilises phylogenetic relationships of pterosaurs and allies to determine their evolutionary history and palaeobiogeography. Specifically, we assembled a supertree, assigned each tip to a geographical area and calculated the potential of dispersal and extend of latitudinal dispersal of pterosaromorphs, compared to close allies.<br>2) Using a newly assembled dataset of Triassic pterosauromorphs we calculated the climate preference of lagerpetids vs pterosaurs, using climate variables calculated for each locality of occurrence. The climatic preference of each group was first assessed using multivariate analyses, and then projected in palaeogeographic maps to assess the distribution of high-suitability areas through time during the Middle to Late Triassic.
Research sample	Unique single occurrence of Triassic pterosauromorphs.
Sampling strategy	For each locality at least one specimen, positively identifiable as either a lagerpetid or a pterosaur was included in the manuscript. Where multiple specimens occurred in the same locality, only one occurrence per group was considered.<br>To avoid redundacy, a single locality was considered if two or more localities resulted indistinguishable due to the resolution of the climate analysis grid. This was the case, unless distinct taxa occurred in the cluster; in that case individual unique occurrences were considered.
Data collection	Occurrence data of pterosauromorphs were collected through the Paleobiology Database (PBDB). This initial search was expanded with literature search, and new occurrences were obtained through fieldwork data from Petrified Forest National Park, Arizona, USA.<br>DF lead the collection of the occurrence data of Triassic pterosauromorphs.<br>Climate data were produced by AF (see methods), and utilised by AAC and EMD for further analyses.
Timing and spatial scale	Our data include pterosauromorphs of the Triassic period. Our study covers the Anisian-Rhaetian stage.
Data exclusions	No occurrence data were excluded except in case it was redundant (e.g., two specimens of the same taxon from the same locality corresponded to a single entry).
Reproducibility	Data and code are available.
Randomization	Datapoints were assigned to one of two groups based on positive morphological criteria (= diagnostic features).
Blinding	n/a

Did the study involve field work? ☐ Yes ☒ No

Reporting for specific materials, systems and methods

We require information from authors about some types of materials, experimental systems and methods used in many studies. Here, indicate whether each material, system or method listed is relevant to your study. If you are not sure if a list item applies to your research, read the appropriate section before selecting a response.

Materials & experimental systems

n/a	Involved in the study
☒	☐ Antibodies
☒	☐ Eukaryotic cell lines
☐	☒ Palaeontology and archaeology
☒	☐ Animals and other organisms
☒	☐ Clinical data
☒	☐ Dual use research of concern
☒	☐ Plants

Methods

n/a	Involved in the study
☒	☐ ChIP-seq
☒	☐ Flow cytometry
☒	☐ MRI-based neuroimaging

Palaeontology and Archaeology

Specimen provenance

Provide provenance information for specimens and describe permits that were obtained for the work (including the name of the issuing authority, the date of issue, and any identifying information). Permits should encompass collection and, where applicable, export.

Specimen deposition

Indicate where the specimens have been deposited to permit free access by other researchers.

Dating methods

If new dates are provided, describe how they were obtained (e.g. collection, storage, sample pretreatment and measurement), where they were obtained (i.e. lab name), the calibration program and the protocol for quality assurance OR state that no new dates are provided.

☒ Tick this box to confirm that the raw and calibrated dates are available in the paper or in Supplementary Information.

Ethics oversight

Identify the organization(s) that approved or provided guidance on the study protocol, OR state that no ethical approval or guidance was required and explain why not.

Note that full information on the approval of the study protocol must also be provided in the manuscript.

Plants

Seed stocks

n/a

Novel plant genotypes

n/a

Authentication

n/a
